# A Precocious Infant

**Published:** 1854-11

**Authors:** 


A Precocious Infant.—Dr. Blake mentioned the following facts: May 11, attended Mrs. H. in her fourth confinement, after a tedious, though not severe labor; the child, a female, unusually vigorous, and weighing, at birth, eleven and a half pounds. On the third day, subsequent to visiting his patient, the nurse asked, "Doctor, do you see our baby laugh?" Incredulous, he crossed the room—but, to his surprise, the infant return d an answering smile to the caresses of the nurse. With a look of intelligence, it turned to the father who stood over it, as he patted its cheeks, and as quickly turned its eyes on Dr. B., when he did the same; and when he stepped aside, its gaze followed him (attracted, he supposes, by his spectacles) until, being out of view, it cried aloud; returning to its side, a smile of pleasure again greeted him, and the cry was once more repeated when he ceased to notice the little one.

Here was unmistakable evidence of pleasurable and sad emotions, and showing the distinct recognition of sensible objects, by an infant as yet scarce sixty hours old. That it was no accidental circumstance, the like manifestations, which were daily noticed afterwards, sufficiently prove.—*Am Jour. of Med. Sciences.*

